# Healthcare professionals and scientists’ collaboration with biobanks: a pilot study on the assessment of knowledge and attitudes toward biospecimen donation

**DOI:** 10.3389/fmed.2025.1497209

**Published:** 2025-02-17

**Authors:** Anastasiia A. Mikhailova, Elena S. Bogomiagkova, Yulia A. Nasykhova, Roman A. Illarionov, Maria M. Danilova, Ziravard N. Tonyan, Vyacheslav B. Chernykh, Ludmila V. Kovalenko, Olesya N. Bespalova, Andrey S. Glotov

**Affiliations:** ^1^D.O. Ott Research Institute of Obstetrics, Gynecology and Reproductology, Saint Petersburg, Russia; ^2^Saint Petersburg State University, Saint Petersburg, Russia; ^3^Research Centre for Medical Genetics, Moscow, Russia; ^4^Budgetary Institution of Highest Education of KHMAO-Yugra “Surgut State University”, Surgut, Russia

**Keywords:** biobank, biobanking, survey, bioresource collection, awareness, medical staff

## Abstract

**Background:**

Difficulties in the biobank progress are often associated with insufficient public awareness, but it is also worth paying attention to healthcare and science professionals who play an important role in the development of this area. This article presents results of the research on awareness toward biobanking and motivation to collaborate among employees of 3 medical and scientific organizations in Russia.

**Methods:**

The anonymous survey was carried out via an online platform. The research included 176 healthcare and science professionals. To assess the differences between the groups the Pearson Chi-square test and the contingency coefficient were used. To find correlations between nominal and interval variables the Eta criterion was applied.

**Results:**

It was found that 88.1% of respondents were aware of biobanking in general, however, 15.0% were not aware of a biobank in their organization or had difficulty in answering this question. The experience of cooperation with biobanks was not particularly extensive – more than half of the respondents (60.3%) indicated that they had never contacted biobanks. 11.9% of participants became donors themselves. 35.0% - suggested to their friends and patients to become donors, while 86.2% were willing to do this in the future. The study showed that the respondents who already had experience working with biobanks, as well as those who themselves act as a donor, rated the importance of their activities higher.

**Conclusion:**

It is important to increase not only awareness but also motivation to cooperate with biobanks and be willing to take on different roles.

## Introduction

A biobank can be defined as a legal entity or a part of a legal entity for long-term storage of large high-quality collections of biospecimens with associated data which follows standard operating procedures and provides materials for scientific and clinical use (1). Modern biobanks appeared at the end of the 20th century and subsequently became an indispensable research infrastructure, involving close interaction between the scientific community, the state, society and economic institutions (2). The advantages of biobanks have been demonstrated for both fundamental and applied research (3–7). In recent years, the number of biorepositories and samples stored in biobanks have grown exponentially all over the world.

Although biological samples have been collected and stored in Russia for more than 100 years, biobanks in the modern sense were established and developed only in the last decade. The vast majority of biobanks, such as “Noah’s Ark”(M.V. Lomonosov Moscow State University, Moscow, Russia), Biobank “Genofond” (D.O. Ott Research Institute of Obstetrics, Gynecology and Reproductology, Saint-Petersburg, Russia), Biobank Sibmed (Siberian State Medical University, Tomsk, Russia), etc. (8) were established based on pre-existing collections at major research institutions and universities through the systematization of samples and infrastructure upgrades. Similar to the largest international professional societies, such as ISBER or BBRI-ERIC (9, 10), the National Association of Biobanks and Biobanking Specialists (NASBIO), established in Russia in 2019, is engaged in the development of cooperation and standardization in the field of biobanking (8). However, as of today (11.01.2025), the NASBIO comprises 33 institutions, 15 of which are located in Moscow and 9 in St. Petersburg – the two largest megacities of the country. This provides an insight into the relatively low prevalence of biobanking in Russia, especially in the provinces, considering the vast size of the country and its population. Despite the fact that the development of genomic research and biobanking is among the priority tasks of state policy (11), this area remains poorly regulated at the legislative level (12), and the rate of biobank development is slower than anticipated. The practice of biological sample donation for the purposes of scientific or medical research has not yet become widespread, the general public and doctors are poorly involved in biobanking processes, which is a serious challenge to the further development of biobanks in Russia.

In order to consistently overcome the low level of engagement in the biobanking process, it seems important to understand the reasons behind this problem for each of the parties involved. The successful development of biobanking requires the engagement of three main parties: biobank staff, potential donors and healthcare specialists. The biobank employers engaged in the functioning of the biobank processes can have a strong impact on public awareness of biobanks by presenting publicly accessible information on various information platforms, mass media publications, and participating in scientific/educational events. Potential donors directly influence the effectiveness of the biobank process, at the same time having limited knowledge about biobanks (13). According to a number of studies, willingness to become a donor depends on a number of factors, ranging from age and education level to the level of trust in the government (13–16). It is noteworthy that even taking into account the above-mentioned factors and limited knowledge about biobanking, most respondents were willing to donate their biological samples (13). A similar pattern was observed in Russia. In accordance with the survey conducted among students of Saint Petersburg State University, the majority of them expressed a willingness to become donors for a biobank, despite a low awareness of such institutions (17). Meanwhile, university faculty are, on the contrary, more informed about biobanks, but they are less willing to donate their biological samples, possibly due to the greater openness of younger individuals to new experiences (18). Prosocial nature of this action motivates individuals to become donors (19). However, despite the declared willingness to become donors, the actual involvement of the Russian population in donation practices remains low. Health system staff, including doctors and medical researchers, act as intermediaries in the interaction between biobanks and donors, facilitating patient recruitment. The researchers suggest that the success of the biobank is based on the support of medical professionals who play a key role in its implementation, attracting patients for donation to the biobank and helping potential donors solve all emerging issues. According to the researchers from Canada, low motivation and insufficient involvement of healthcare professionals reduce the effectiveness of donor recruitment and can even have a detrimental impact on the development of both individual biobanks and the biobank industry as a whole (20). Most studies show a positive attitude of healthcare specialists toward biobanks, but it is necessary to emphasize some doubts. For example, the authors noted the discomfort that doctors experience when giving consent to a patient (21), the need to improve the privacy protection system (22). According to the Australian study, only half of practitioners are willing to participate in collecting biological samples from their patients for a biobank (23). In Russia, the study by Antonova N. and Eritsyan K. suggests that a high level of trust in a specific physician may facilitate favorable decisions regarding biobank donation, thereby emphasizing the importance of involving medical personnel in the biobanking process (19). It can be assumed that studying the awareness of biobanks of all the parties involved and their willingness to collaborate with biobanks can contribute to the identification of hitherto obscure issues arising in the practice of donating biological samples for scientific purposes.

In Russia, the body of research on understanding attitudes toward biobanks is relatively limited (24). Existing studies focus primarily on various accessible social groups, such as students and university staff (17, 18), or individuals who have prior donor experience (19). Recognizing that the success of biobanking is contingent not only upon public awareness and individual willingness to become donors but also on the engagement, interest, and motivation of medical and scientific professionals, we decided to investigate this specific group in our research. This focus is crucial for developing a comprehensive understanding of the factors that influence biobanking initiatives.

The study aims to assess the awareness of biobanks among employees of three scientific and educational institutions in Russia and to analyze their willingness to collaborate with biobanks, including the donation of their own biological material. Given that there was no reliable sociological information on the issues considered at the moment when the study was performed, this study is mainly of a search and descriptive nature.

## Materials and methods

### Participants and recruitment

The study was conducted in three research and educational organizations that comprise the Network Russian Center of Bioresource Collection “Human Reproductive Health” which aims to collect a biobank of biological samples from patients with reproductive disorders, severe obstetric pathologies, and genetic diseases for further research:

1. D.O. Ott Research Institute of Obstetrics, Gynecology and Reproductology, Saint-Petersburg (institution 1) - the federal scientific and medical institution in the Russian Federation in the field of obstetrics and gynecology.
2. Research Centre for Medical Genetics, Moscow (institution 2) - the federal scientific center in the country in the field of medical genetics and human genetics.
3. Budgetary Institution of Highest Education of KHMAO-Yugra Surgut State University, Surgut (institution 3) - the first higher educational institution in the KHMAO-Yugra region (Siberia).

These organizations were selected for the study because from 2021 to 2024 they served as the basis for the implementation of a scientific project titled “Multicenter Bioresource Collection “Human Reproductive Health” (Agreement No. 075–15–2021-1058 dated September 28, 2021),” which included, among other activities, the collection of biological material samples from various patient groups. A condition for participation in the project was the establishment of a collaboration among scientific centers, which necessarily included not only leading large institutions but also regional organizations, as this is essential for the advancement of science in provincial areas. The Surgut State University was included in the project as a regional center.

A total of 176 people took part. The survey was carried out via an online platform. A link to Google Form with the questionnaire was distributed among the employees of three organizations. Participation in the survey was voluntary. All employees who completed the questionnaire properly were included in this study. The questionnaire was designed in such a way that the time required to complete it did not exceed 10 min.

### Survey development

A questionnaire containing 28 questions on the topic of the study was designed for the survey. The survey was completely anonymous, but at the end of the questionnaire there was an email field, which was left solely at the respondent’s request for feedback.

The questions were developed taking into account the current challenges facing the biobanking industry in the Russian Federation, as experienced by colleagues in practice. Moreover, the findings of research on related topics, which cover a variety of factors that have a direct impact on the development of biobanks in a specific geographical region, were utilized in the formulation of the questionnaire. The following keywords were used in the search of literature and relevant links in PubMed: “biobank,” “biobanking,” “collection,” “attitude,” “opinion,” “researchers,” “medical staff,” and “awareness.”

The initial section of the questionnaire encompassed general socio-demographic information, including gender, age, level of education, profession, job position, and place of employment. Furthermore, a dichotomous variable comprising two categories, “medical” and “non-medical,” was introduced to reflect the respondents’ areas of specialization. Those with a medical education and license to practice medicine in Russia were classified as “medical,” while the remaining respondents were designated as belonging to “non-medical” specialties. This was followed by an assessment of biobanking awareness (information sources, knowledge of biobank activities etc.), as well as own experience of collaboration with biobanks. The respondents’ attitude toward cooperation with biobanks, as well as their willingness to donate and motivation to involve patients in donation, were then investigated. The focus was on both the experience of applying to biobanks and the experience of donations and recommendations. Furthermore, we assessed the respondents’ understanding of legal and ethical considerations in the field of biobanking. The final section of the questionnaire was aimed to explore the demand for biobanks and stored collections among the respondents, which may help to identify the problems and challenges hindering development of this field in Russia.

The questionnaire consisted mainly of closed questions. The respondent expressed their opinion by choosing one or more answer options. In some instances, the respondent had the chance to express their option. Ordinal and nominal scales were utilized to assess the opinions of the respondents.

### Statistical analysis

The data collected in the questionnaires was verified and checked for completeness, quality, and consistency and exported to the statistical packages SPSS (Version 23.0). The results were presented as descriptive statistics. Since the signs of interest to researchers were measured mainly on nominal scales, the assessment of the differences in the distribution of variables among groups consisted of applying the Pearson Chi-square test and calculating the contingency coefficient (Pearson). The corresponding values of the conjugacy coefficient are indicated in the text. To find correlations between nominal and interval variables the Eta criterion was applied, so it was marked in brackets additionally. *p*-value <0.05 was considered significant. Although the majority of the relationships we identified were moderate, they enabled us to make important assumptions about the actual relationships between the variables under study.

### Ethical considerations

The study was approved by the Institutional Ethics Committee of D.O. Ott Institute of Obstetrics, Gynecology, and Reproductology (protocol #113 dated November 18, 2021).

Additional written informed consent was not required for study participants, since the very fact of completing the survey was equivalent to consenting to participate.

## Results

### Socio-demographic characteristics

The study involved 176 employees from three organizations (see Table 1), of which 52.8% of respondents were from Institution 1, 14.8% — from Institution 2, and 32.4% — from Institution 3. 83% of all respondents were women and 17% — men. The sample mostly consisted of the age groups from 26 to 35 years (33.7%) and from 36 to 50 years (40.0%). Most respondents (61.4%) had a medical education. Candidate of Sciences (PhD) and Doctor of Sciences degrees were held by 29.0 and 13.1% of respondents, respectively. More than half of respondents were researchers (51.1%), the share of doctors was 21.6% of the sample.

**Table 1:** Socio-demographic characteristics of respondents.

Socio-demographic characteristics of respondents	Population, *N* (%)
Gender (*N* = 176)	
Male	30 (17.0)
Female	146 (83.0)
Institution (*N* = 176)	
Institution 1	93 (52.8)
Institution 2	26 (14.8)
Institution 3	57 (32.4)
Age (*N* = 175)	
18–25	11 (6.3)
26–35	59 (33.7)
36–50	70 (40.0)
51–65	27 (15.3)
> 66	8 (4.5)
Profession (*N* = 176)	
Medical	108 (61.4)
Non-medical	68 (38.6)
Education level (*N* = 176)*	
Bachelor	4 (2.3)
Master	14 (8.0)
Specialist	63 (35.8)
Candidate of sciences (PhD)	51 (28.9)
Doctor of sciences	23 (13.0)
Secondary general education	1 (0.6)
Secondary vocational education	20 (11.4)
Job position (*N* = 176)	
Researcher	90 (51.1)
Laboratory assistant	20 (11.4)
Physician	38 (21.6)
Nursing staff	9 (5.1)
Postgraduate, master’s student, student	3 (1.7)
Administrative staff	16 (9.1)

*Professional education in Russia consists of several levels: secondary general education; secondary vocational education; first cycle university education – bachelor’s degree; second cycle university education – master’s degree and specialist degree (only for certain medical and engineering specialities); the highest scientific qualification personnel training: Candidate of Sciences (equivalent to PhD degree) and Doctor of Sciences (Higher Doctorate).

### Assessment of awareness of biobanks and their activities

First we analyzed the respondents’ awareness about the biobanks and their goals and objectives (see Table 2). According to the data obtained, the majority of the study participants were familiar with the concept of biobank (88.1%). Although 2.8% of respondents were undecided, they were still included in the further survey. Thus, the main study group consisted of 160 people.

**Table 2:** Awareness of biobanks.

	Yes, *N* (%)	No, *N* (%)	Do not know, *N* (%)
Have you ever heard of biobanks? (*N* = 176)	155 (88.1)	16 (9.1)	5 (2.8)
Do you know that there is a biobank in your organization? (*N* = 160)	136 (85.0)	15 (9.4)	9 (5.6)

It was noted that with the increasing age of respondents, there was a corresponding increase in their awareness of the concept of biobanks (0.283, *p*-value = 0.054). Thus, 63.6% of respondents aged 18 to 25 heard of biobanks, whereas the rate of such participants in the age group over 50 years was 100%.

**Figure 1:**
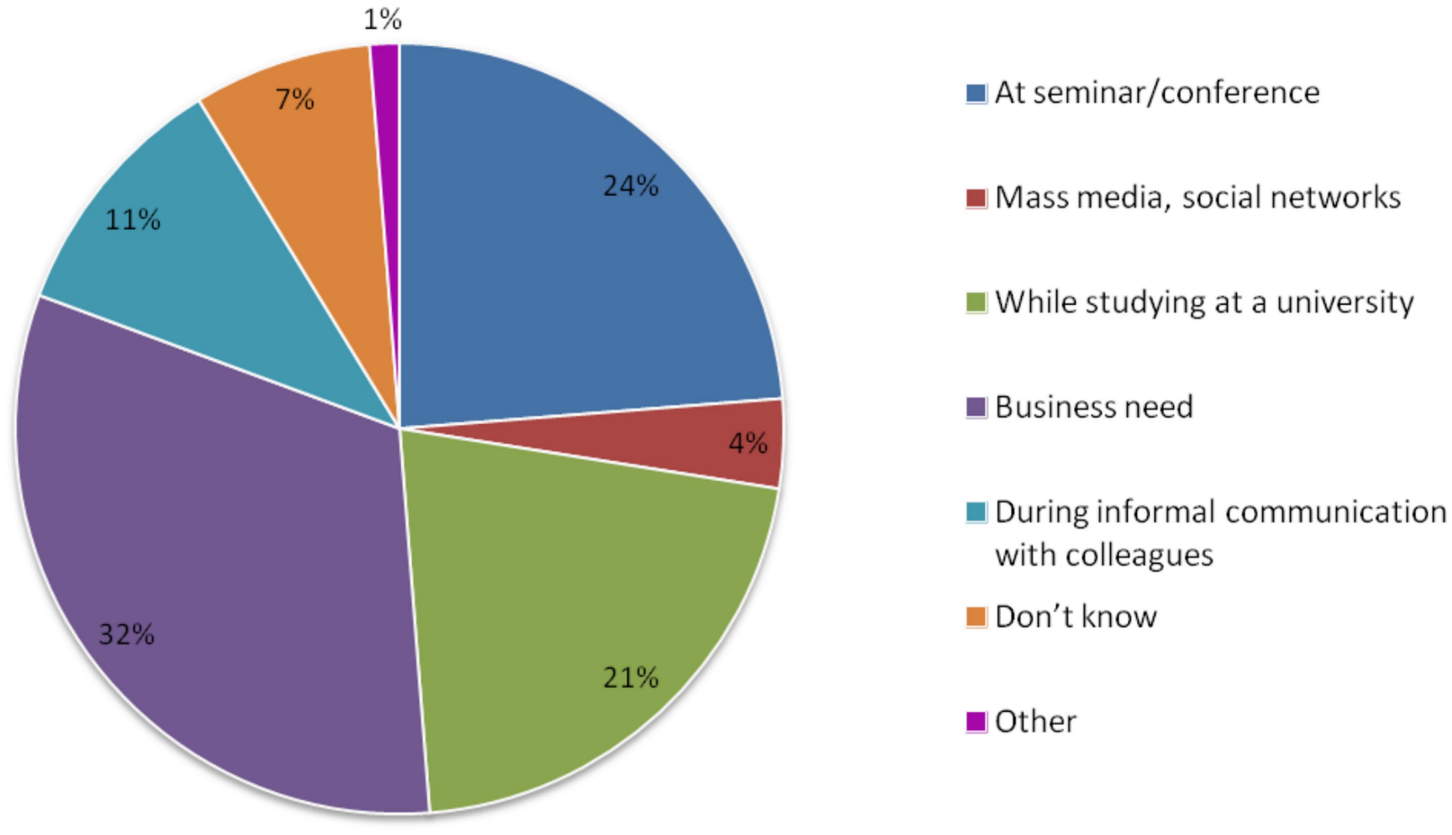
Respondents’ answers to the question: “How did you hear about biobanks?”, *N* = 160.

The respondents indicated that seminars and conferences (23.8%) were the primary source of information about biobanks, followed by business needs (32.0%) (see Figure 1). Therefore, the primary sources of information about biobanks were more closely associated with professional activities.

**Figure 2:**
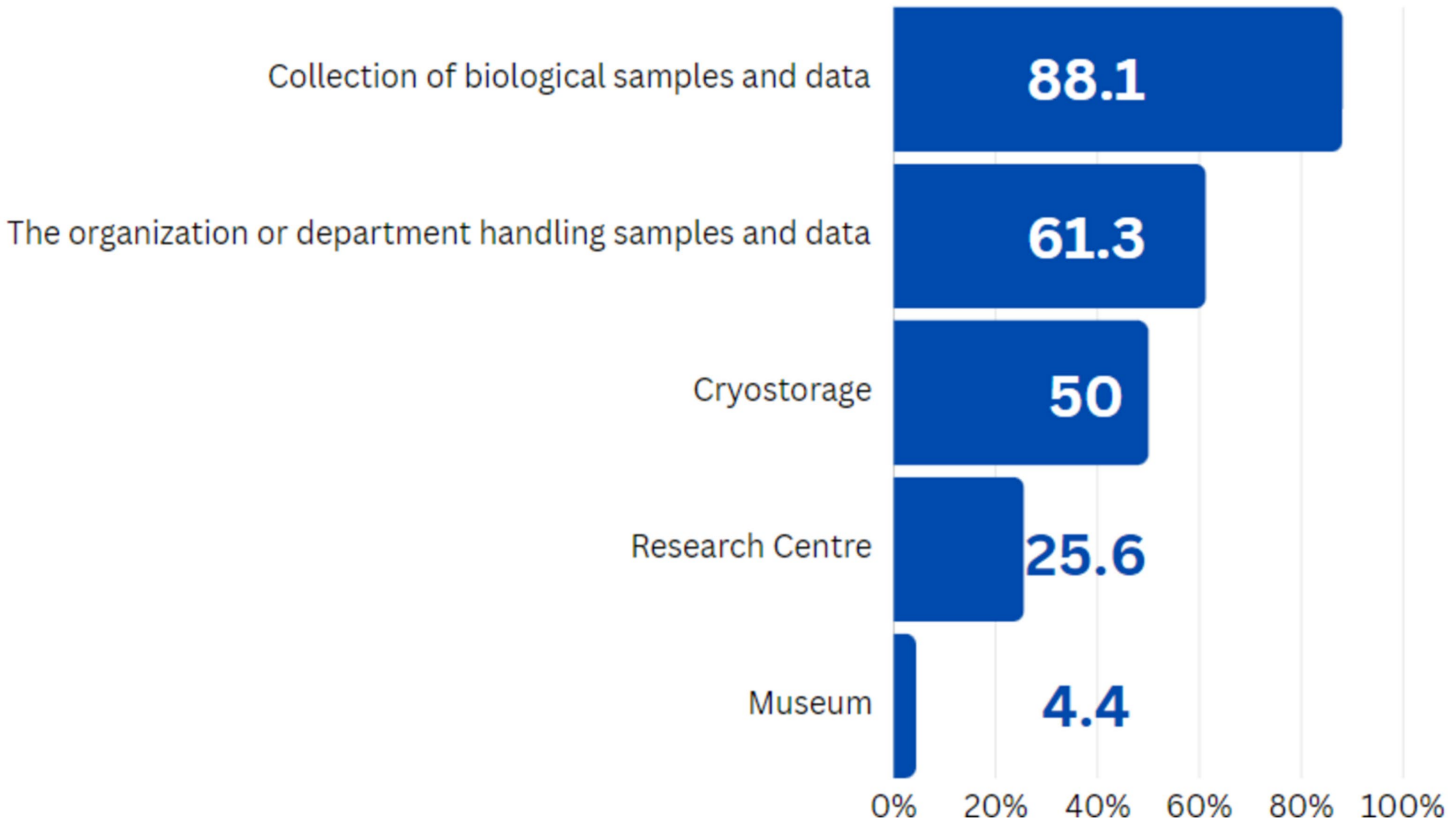
Respondents’ answers to the question: “What is a biobank?”, *N* = 160.

In addition to general awareness, it was also important to clarify how well the respondents were familiar with the various aspects of the functioning of biobanks. So, just over half of respondents (61.3%) understood biobank as an organization or division that engages in the collection, storage, and analysis of biological samples and data, which corresponds to the National Biobanking Guidelines (8). The majority of respondents (88.1%) indicated that biobank is a «collection of biological samples and data». Since respondents were permitted to choose multiple answer options, it is probable that some of the responses selected reflected their subjective interpretation of the fundamental nature of biobank (see Figure 2). Furthermore, approximately 50% of respondents identified biobank as cryogenic storage. It is noteworthy that most of the respondents considered biobanks as repositories for biological samples, rather than as platforms for scientific research and experimentation.

Next, we investigated the knowledge about the ways of receiving biological samples into the biobank and interaction with potential donors.

84.4% of respondents believed that the donor specifically submitted samples to the biobank, 60.6% believed that the remaining samples could be transferred to the biobank after the completion of other procedures. Of the respondents who answered the question (*N* = 160), 47.5% selected both responses.

Three quarters of respondents (76.3%) agreed that informed voluntary consent from a donor was required for the submission of samples to the biobank, however, 18.2% of respondents believed that it was generally possible or possible in some cases to ignore a voluntary consent. Interestingly, slightly more than half of the respondents (50.7%) believed that the donor deserved something in return for donating biomaterial to the biobank. 69.1% of them suggested that compensation could be represented by some services, for example, a doctor’s consultation or some kind of laboratory test, while 70.4% thought that the donor was entitled to be informed of all the results of scientific research in which their sample was included, and about half of the respondents (46.9%) named money as a reward.

The improper interpretation of various aspects of donation when answering questions about the ethical aspects of biobanking can be seen as a manifestation of insufficient legal and ethical regulation in this field of scientific activity. Legal documents do not clearly define and enshrine the rights and obligations of the parties (donors and biobanks). This may lead to a degree of hesitation and doubt among a number of specialists, given that the legal field of biobanking in Russia is not yet specifically regulated, and this may be one of a major impediment to the development of this industry.

Despite the general awareness of biobanks, almost 15.0% did not know about the presence of a biobank in their organization or found it difficult to answer this question.

The data obtained may indicate, on the one hand, insufficient work on informing employees about the activities of biobanks in their organizations, and, on the other hand, possibly insufficient motivation of employees themselves in the search for information of this kind. A greater proportion of respondents aged 18–39 (78.5%) indicated that they were somewhat less informed about the presence of a biobank in their organization than respondents over 40 years (91.3%) (0.197, *p*-value = 0.041).

We asked to evaluate the availability of information about the organization’s biobank on a scale from 1 to 3. The average score was 2.2. It is noteworthy that the average information availability rating was higher for those who had previously applied to biobanks (2.4), and for those who intended to do so in the future (2.5), compared to those who did not register and did not intend to do so (1.9) (Eta coefficient 0.4, *p*-value = 0.000). These differences can be interpreted as follows: specialists who have collaborated with biobanks and are prepared to do so in the future are more aware of their activities and may have independently searched for information about biobanks, which may contribute to their greater satisfaction with the availability of such information. Those who have never applied to biobanks and do not intend to do so in the future rate the availability of information as below average, since they have no motivation to search for it.

**Figure 3:**
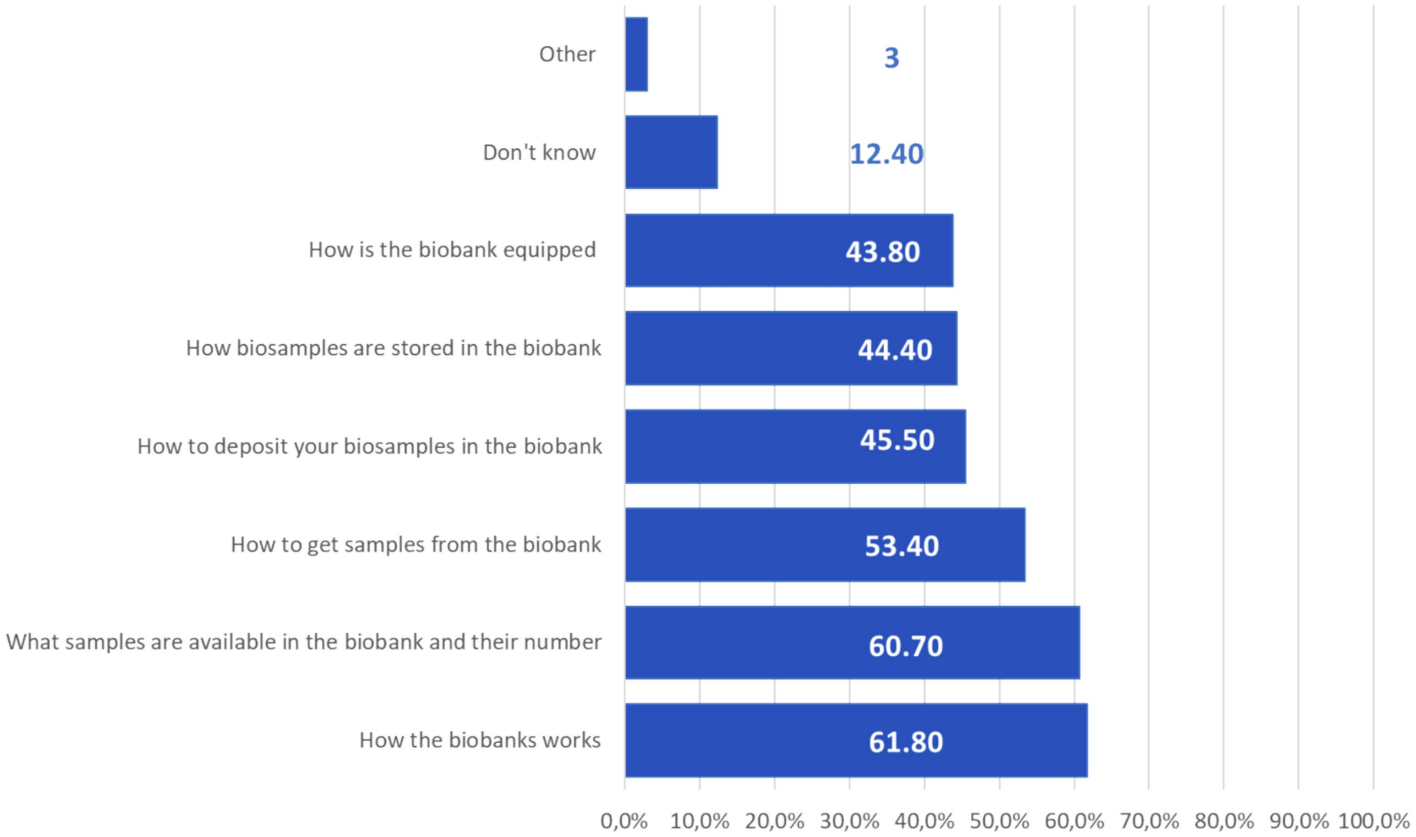
Respondents’ answers to the question “What information would you like to learn more about the biobank in your institution?”, *N* = 176.

In the question that was available to all participants of the study, regardless of how they answered earlier, it was suggested to note all the points of interest that they would like to know about the biobank at their institution. Among all respondents, 61.8% of respondents felt the need for additional information about how biobanks worked, 60.7% were interested in what samples were in the biobank, their number, and 53.4% would like to know how to get samples from the biobank (Figure 3).

Consequently, despite the articulated awareness, specialists still require additional information about the biobank’s activities within their organization.

### Researching the experience of interaction and willingness to cooperate

More than half of the respondents (60.3%) indicated that they had never contacted a biobank. Among those who were aware of the biobank in their organization (*N* = 136), a quarter (26.5%) had experience of interaction with it, and another third (29.4%) were going to do so in the future. It should be emphasized that a third of the respondents (30.9%) did not previously request samples or services from a biobank or plan to do it in the future. However, it should be noted that not all employees of the organizations studied have the need for biobank services in their professional activities.

Among the respondents who were aware of the biobank in their organization, a third (30.1%) indicated that samples from the biobank were not available to them, and 32.4% found it difficult to answer this question. This attitude may also discourage people from applying for samples in biobanks.

Interestingly, the availability of samples was rated higher by respondents who previously had experience in work with biobanks (55.6% of them considered that samples were fully available). Among those who had not yet interacted with biobanks but planned to do so in the future, 22.5% believed that samples were fully available. Among respondents who had no experience of work with biobanks and did not plan to do so in the future, only 2.4% believed that samples were fully available, and 50.0% believed that they were not fully available. The differences were statistically significant (0.633, *p*-value = 0.000).

The majority of respondents who were aware of the biobank in their organization (93.4% - the sum of the answers “yes” and “rather yes”) were ready to participate in its further development.

In addition to experience in requesting samples, we were also interested in personal experience of donating their materials, as well as inviting their patients and relatives to become donors. We assumed that this activity can also be considered as experience of cooperation with biobanks.

One third (35%) of respondents indicated that they recommended their friends and patients to become donors, while only 11.9% donated their biological material themselves (Table 3).

**Table 3:** Experience of interaction with biobanks.

	Yes, *N* (%)	No, *N* (%)	Do not know, *N* (%)
Have you ever donated your biological material to a biobank? (*N* = 160)	19 (11.9)	137 (85.6)	4 (2.5)
Have you ever offered your friends or patients to participate in the donation of biological material to a biobank? (*N* = 160)	56 (35.0)	100 (62.5)	4 (2.5)
Have you ever requested samples or services from your organization’s biobank? (*N* = 136)	36 (26.5)	82 (60.3)	18 (13.2)

80.4% (the sum of the answers “definitely yes” and “rather yes”) of the study participants expressed their willingness to donate their biological material to the biobank in the future and 86.2% (the sum of the answers “definitely yes” and “rather yes”) of respondents were ready to offer their friends or patients to donate their biomaterial to biobank (see Table 4).

**Table 4:** Willingness to cooperation with biobanks.

	Definitely yes, *N* (%)	Rather yes, *N* (%)	Rather not, *N* (%)	Definitely not, *N* (%)
Are you ready to donate your biological material to a biobank in the future? (*N* = 160)	64 (40.0)	71 (44.4)	22 (13.8)	3 (1.8)
Are you ready to offer your patients or friends to donate their biological material to a biobank? (*N* = 160)	61 (38.1)	77 (48.1)	19 (11.9)	3 (1.9)
Are you ready to take part in the development of a biobank in your organization? (*N* = 136)	73 (53.7)	54 (39.7)	8 (5.9)	1 (0.7)

It is interesting to note that the individual experience of biomaterial donating or the willingness to donate in the future was statistically related to the willingness to recommend this to patients. Thus, respondents who donated themselves were much more likely than those who did not to offer patients to donate their material (0.383, *p*-value = 0.000), and were willing to be donors in the future (0.324, *p*-value = 0.004). In addition, those who planned to donate their material in the future recommended patients to do it more frequently (0.420, p-value = 0.000) and were more willing to offer it in the future (0.713, *p*-value = 0.000). Those who already recruited patients to donate their biomaterial were more likely to do so further (0.407, *p*-value = 0.000). Noteworthy, some of the highest coefficients of conjugacy were recorded for these indicators in this study. We therefore can make a confident assumption that the personal experience of an employee (both the delivery of their biomaterial, and the willingness to do so in the future) is generally related to how ready they are to offer the patient to donate the biomaterial or have already done it. That is, not only awareness of employees of the biobanks’ activities, but also personal experience of involvement in their work as a donor of biomaterials are important for more effective work with patients as potential donors of biobanks.

We would like also to point out another interesting aspect: a statistically significant correlation was found between the experience of requesting for samples to the biobank (respondents had previously requested to the biobank for samples) and the experience of involving friends and relatives in donation (0.408, *p*-value = 0.000) and the readiness to do it in the future (0.468, *p*-value = 0.000). In addition, those who applied for samples to the biobank were slightly more likely to have already submitted samples themselves (0.359, *p*-value = 0.003) and were ready to do so in the future (0.436, *p*-value = 0.000). Thus, we emphasize that it is the personal experience and professional interest of specialists that serves as the basis for recruiting patients and acquaintances as donors to biobanks.

### Identification of problems and obstacles to the development of biobanking in institutions

Furthermore, the respondents were asked to indicate what possible problems and shortcomings they believe are currently present in biobanking, both within their organizations and in this field of activity in general.

**Figure 4:**
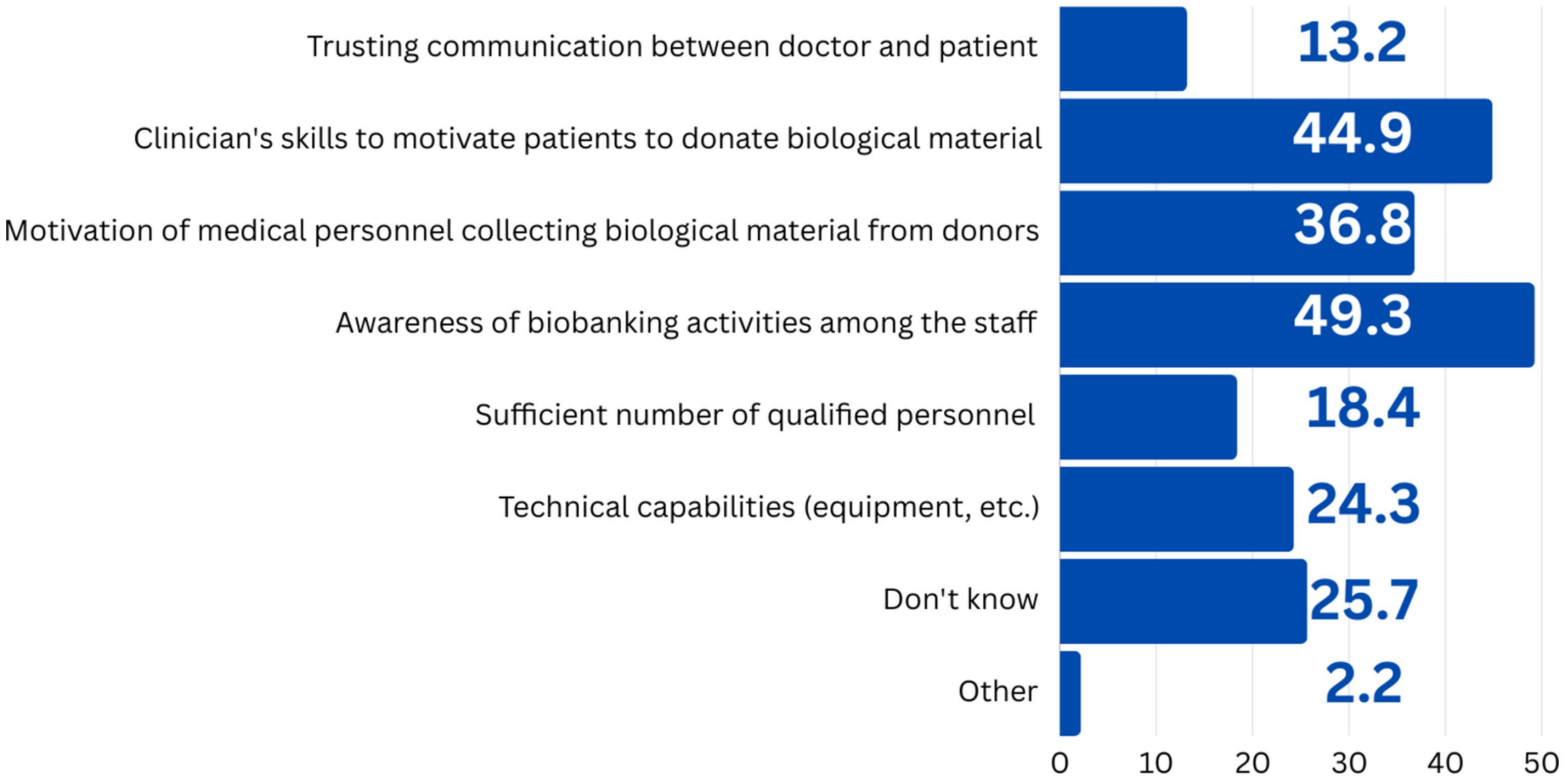
Respondents’ answers to the question “What is missing for the development of your organization’s biobanks?”, *N* = 136.

The study revealed that employees were not adequately informed about the work of biobanks within their organization, as evidenced by their responses to other questions. Thus, almost half (49.3%) of respondents declared that the staff of the institution lacks awareness of the biobanks’ activities for the development of biobanks; the next most popular answer (44.9%) was that doctors lack the skill to motivate patients to donate biological material. 36.8% were sure that there was a lack of motivation of medical personnel collecting biological samples. It should be noted that significantly fewer choices were given to options that characterize communication between doctor and patient, the level of staff qualifications, and the lack of technical capabilities of the organization (Figure 4). The emphasis on the dearth of motivation among staff is corroborated by the findings on the minimal engagement of respondents in collaboration with biobanks.

**Figure 5:**
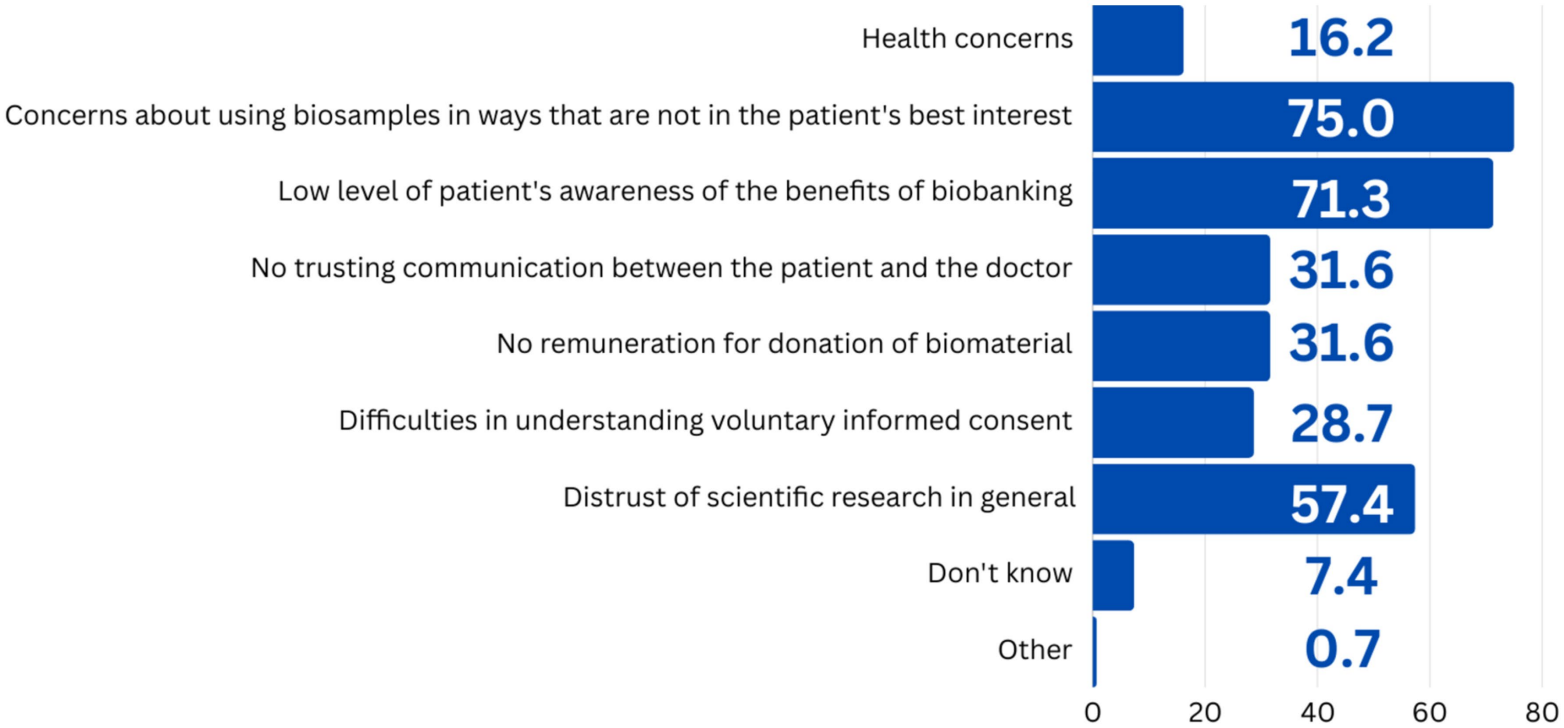
Respondents’ answers to the question “What, in your opinion, are the obstacles to consent to the donation of biological material from donors/patients?”, *N* = 136.

As the most important reason hindering the development of an entire industry of biobanks, respondents cited the fear of potential donors of their biomaterial misuse (75.0%). Low public awareness of biobanks was cited by 71.3% of respondents. Slightly more than half (57.4%) believed that distrust of scientific research in society as a whole was an obstacle (Figure 5). Thus, the assessment of the most significant obstacles to the development of biobanks and the participation of the population in donation reflect the lack of ethical and legal regulation of the activities of biobanks. According to respondents, since it is unclear how the delivery of biological samples is regulated, why such samples are used, etc., the population is not ready to act as donors and cooperate with biobanks.

Thus, before informing the public, it is necessary to develop statutory documents that regulate the activities of biobanks and the procedure of donation. It is necessary to understand what to inform about in order to avoid the emergence of public fear and apprehension.

## Discussion

Attention to the professional community is a novel aspect of the Russian context, given that research predominantly focuses on public attitudes toward biobanking. The objective of this study was to assess the awareness of employees of the Network Russian Center of Bioresource Collection “Human Reproductive Health” toward biobanks and their willingness to interact with them. Additionally, we aimed to investigate the experience of collaborating with biobanks and the difficulties that arose. In this study 176 people were included, among them representatives of various specialties, age groups, as well as those with different levels of education.

When assessing general awareness of biobanks, their goals and objectives, there were no statistically significant differences depending on the education and specialty of the respondents. There were also no significant differences between the institutions studied. However, the respondents of older age groups were more knowledgeable about both the activities of the biobank as a whole and how it operates within the organization compared to younger ones. Thus, 63.6% of respondents aged 18 to 25 heard of biobanks, whereas the rate of such participants in the age group over 50 years was 100%. We suppose that this can be explained by the fact that the older generation (over 50 years old) often participates in scientific and practical seminars and conferences in their professional activities where the topic of biobanking has often been discussed recently. It is plausible that biobanking is paid little attention within the framework of university education. In some other studies (25), an opposite trend can be observed: younger professionals are better informed about biobanks compared to older age groups. Thus, in Russia, awareness may be more closely related to experience and presumed age, whereas higher awareness among younger groups likely indicates their greater openness to innovation.

Our analysis of responses regarding sources of information about biobanks yielded consistent results. The most common answers were the following: at seminar/conference (23.8%) or business need (32.0%) (Figure 1). These sources serve as the primary means for professionals across various occupational groups and sociocultural contexts to obtain information about biobanks, as indicated by findings from different countries (23, 25, 26). In contrast to the general population, which primarily gathers information about genetic research and biobanking through mass media (27), medical professionals rely on both formal and informal professional communication as their key channels of information used at specially organized events as well as in the workplace. This underscores the importance of enhancing this mode of information dissemination.

Our findings indicated that the awareness of biobanks was significantly greater for Russian medical professionals (88.1%) than biomedical professionals surveyed in Côte d’Ivoire (56.6%) (26), Saudi Arabia (34.0%) (28), Morocco (37.5%) (25), Australia (35.8%) (23), and Egypt (65.5%) (29). However, unlike foreign studies that focus on medical professionals of various specialties in general, the present study involved employees from organizations that already have biobanks, which likely affected their higher levels of awareness regarding biobanking.

However, not all respondents were able to correctly characterize the essence of biobanks’ activities. According to our survey results, the most frequent response was to understand the biobank as a “repository” or “collection” of biological samples. Most respondents did not know that in addition to the objectives of storing and distributing biological samples, the biobanks often perform a function in accumulating and processing large-scale data (including omics data), donor recruiting, developing documents and procedures to ensure management and quality control, as well the biorepositories can directly participate in biomedical scientific research. The collection and storage functions were likely considered as the main tasks in the activity of biobanks.

Approximately 15% of those who had heard of biobanks did not know about its work in their organization or found it difficult to answer this question, which is a fairly high indicator. This further emphasizes the need for activities aimed at raising awareness of biobanks’ functions within organizations. Of those who knew about the work of the biobank, a quarter (26.5%) of respondents already applied to the biobank, another third (29.4%) planned to do it in the future, a third of respondents (30.9%) neither applied nor planned to do it in the future. Thus, not all respondents demonstrate willingness and motivation to cooperate with biobanks. Respondents with actual experience of collaboration with biobanks estimated the availability of samples and information on biobanks’ operations slightly higher than those who never applied for samples nor planned to do so. Thus, personal experience of collaboration with biobanks affects the evaluation of their activities.

It is noteworthy that, unlike several other studies, we focused not only on attitudes toward potential collaboration with biobanks but also on the actual experience of collaboration, donation, and the involvement of relatives and patients as donors.

Some limitations identified in previous studies have been addressed in the present research. Most studies have focused solely on assessing the hypothetical willingness to support biobanks; however, evaluation of the actual participation may yield different, probably lower, figures. A novel aspect of this study is our inquiry into not only the willingness and prospects for collaboration with biobanks (including readiness to recruit donors and to become donors themselves) but also the existing experiences of collaboration. The importance of physician involvement in patient recruitment was also demonstrated in the study conducted by Fradgley et al. (30).

Our findings suggest that not merely potential willingness but rather existing experience significantly influences the attitude toward biobanking.

In this study, we understood cooperation with biobanks not only as willingness (or previous experience) of specialists to use biospecimens in scientific work, but also as donor experience and willingness to motivate or involve friends and patients in this practice. In this regard, it should be noted that those who had experience in working with biobanks, compared to those who did not have, were more likely to donate samples themselves and plan to do so in the future. They were also somewhat more likely to recommend their patients and friends to become donors or had already done so in the past. The correlation between the willingness to become donors and involvement in medical research was also identified by researchers in Saudi Arabia (28).

In addition, statistically significant correlations were found between the respondents’ own experience in donation and the involvement of friends and patients in donation, as well as their willingness to do so in the future. Respondents who themselves had donated biological samples at least once were much more likely and willing to offer this to their friends and patients. However, this practice was typical for only a small part of the respondents. We came to the conclusion that a personal example for many potential donors is a factor that contributes to making a decision. It is important not only to inform employees about biobanking but also to motivate them for cooperation at different levels of interaction.

It is also noteworthy that the majority of those who were aware of biobanks (84.4% of respondents indicating “yes” or “rather yes”) expressed a willingness to become donors themselves, and 86.2% expressed a readiness to encourage relatives and friends to become donors. Similar results were obtained in a study in Morocco (25), with 82.9% of the participants willing to donate their biological samples to biobanks and 82.8% supporting the recruitment of patients into biobanks, while only 37.5% of respondents said they knew what a biobank was. In Colombia, 96% of the surveyed specialists of various profiles expressed their willingness to become donors, while about half of the sample knew what a biobank was, and less than 3% had a clear understanding of it (31). In these cases, the desire to become a donor and help attract patients was accompanied by a low level of awareness, i.e., when expressing willingness, respondents probably did not always understand what they were talking about. At the same time, this willingness was accompanied by significant uncertainty among respondents in some other studies – in the research performed by Abdelhafiz and colleagues 39% were unsure about their readiness to become donors and to involve their relatives in this process (29). Additionally, 49% of surveyed individuals in Australia were uncertain about their willingness to assist in recruiting donors (23), and another 32% of respondents in Saudi Arabia (28) expressed a desire to become donors. In Scotland (21), 40% of the surveyed specialists noted that it would be uncomfortable for them to engage patients in donation. Thus, we emphasize that employment in organizations associated with biobanks may cultivate more favorable attitudes toward professionals’ willingness to become donors themselves and to encourage their relatives and patients to participate in this process. This phenomenon distinguishes them from the attitudes observed among the broader population of medical professionals. In Russia, the high levels of respondents’ willingness to become donors and to promote donor participation among relatives and patients are accompanied by a relatively elevated level of awareness regarding biobanks.

In general, respondents noted that information on the work of biobanks in their organization was available, and therefore it can be assumed that access to it depends on the motivation of specialists to cooperate with biobanks. At the same time, both specialists with experience in applying to biobanks and those without it expressed interest in learning more about the work of biobanks. 61.8% of all study participants felt the need for additional information about how biobanks work, 60.7% were interested in what samples the biobank contained and how much was available, 53.4% would like to know how to get samples from the biobank. The lack of information regarding biobanking is a concern acknowledged by the majority of participants across various studies, irrespective of the country (26, 29). According to other studies, the most frequently requested information about biobanks covers such general aspects as the goals, objectives, and organizational structure of biobanks (25–30), which is generally consistent with the results of our study.

As stated by the respondents, staff members’ lack of awareness is the primary obstacle to the advancement of biobanking initiatives within the organizations (49.3%). The next most popular reported reasons are the insufficient skills of doctors to encourage patients to donate biological material (44.9%) and the lack of motivation of medical personnel in collecting biological samples (36.8%). Once again, we would like to point out that in addition to awareness issues, the specialists themselves consider motivational aspects a significant obstacle to development of biobanks. At the same time, the lack of information about the benefits of biobanking, but from the population, ranks among the top 3 most popular impediments to consent to the donation of biological material from donors or patients (71.3%).

The current study has demonstrated that it is important for the development of biobanks not only to raise both public and medical professionals’ awareness of the work and benefits of biobanks, but also to involve the latter in the work of biobanks, both as researchers and donors, and those who attract and motivate patients. Increasing the motivation of employees themselves for cooperation with biobanks and their ability to encourage patients to become donors (including by personal example) can be key drivers for the development of this area of research.

The findings indicate that despite a relatively high level of awareness regarding biobanks, the proportion of respondents with actual experience in collaboration with them remains low. This underscores the need for the industry’s development to extend beyond mere awareness among specialists; it also necessitates motivation for collaboration and positive personal experiences related to working with biobanks and donations.

It is often not interesting for practitioners to collaborate with biobanks, since they are not researchers, and therefore do not need to collect samples or patients to prepare scientific research. In such a situation, the need to offer their patients to become donors, explain to them how to fill out an informed voluntary consent, and organize the delivery of biomaterial samples may be considered by them as an additional burden.

So, in addition to information about biobanks, which the study participants noted as lacking, it is necessary to motivate specialists and develop special skills for motivation and communication with potential donor patients. This can be achieved through conferences, interinstitutional cooperation, and the creation of interdisciplinary teams. Thus, it is necessary to form the interest of professionals in the development of biobanks, which is possible by elaboration of a mechanism for additional material and non-material incentives for attracting donors.

The study demonstrated that personal donation experience is essential and requires medical professionals to become donors themselves. In a situation where the employees of organizations with biobanks rarely become donors themselves, it is illogical to expect greater engagement from a population that is generally less informed and less interested. Even biobank staff members, who are expected to be highly informed and motivated, are rarely willing to act as donors and recruit donors themselves. Probably, in this case, it is necessary to encourage research activities, form research teams, and elaborate a system of grants and projects. In general, the management of organizations should be interested in the development and promotion of biobanks as well as in the involvement of employees in their activities.

When discussing potential donors, it is important to mention that blood donation in Russia is regulated by legislation (32) that defines the principles of donation, the rights and obligations of donors, as well as social support measures, such as extra days off and meal allowance. Donors also undergo a medical examination before donating blood. However, these measures do not apply when providing samples for biobanks, and any remuneration or compensation is dependent on the specific biobank, which may or may not offer such incentives. Personal experience indicates that potential donors are more willing to agree to sample donation when offered laboratory tests. Physicians find it easier to motivate patients to participate in sample donation for biobanks if there is an opportunity to provide compensation or services in return. If there is no way to guarantee material compensation for biological material donation, it can be ensured that non-material benefits are obtained (f.e., an additional consultation with a physician, access to the results of all studies for which the sample was used, or emphasizing the contribution to the advancement of science). The barriers related to lack of knowledge, regulatory frameworks and social guarantees for donors of biological samples need to be overcome. The success of biobanks depends on the institutional trust of society, which requires transparent information about the procedure itself, the use of samples and their significance for science. Educational initiatives and dialogue between the scientific community, government agencies and society will help to increase the participation of citizens in donation for scientific purposes and make a significant contribution to the development of medicine as a whole.

### Limitations

Among the limitations of this study, it should be noted first that the sample size is small and thus the results of this study cannot be fully extrapolated to represent the professional community. Since the research team lacks access to comprehensive data on the entire population of medical professionals, including those within the organizations under study, it is challenging to determine how well our sample represents the broader population in terms of socio-demographic characteristics. However, we observed that our sample did not reveal any statistically significant differences in socio-demographic and institutional characteristics, which was unexpected. These issues clearly warrant further investigation and careful analysis. Herewith, this study is the first one in Russia that examines the attitudes and experiences of medical professionals working in organizations with biobanks. The study was conducted by an interdisciplinary team including geneticists, biobank specialists, and sociologists. It can be assumed that the problems and difficulties of the biobanking industry, identified in this example, may be even more acute in smaller organizations and generally characterize the perception and attitude toward biobanking by the Russian professional community. Secondly, the fact that the survey was conducted using administrative resources may affect the social desirability of the responses. But, contrary to this, a range of difficulties and contradictions characterizing the attitudes of the professional community toward biobanking and biomaterial donation was revealed.

Although the implemented research can be described as a pilot study, it explicates important findings and allows us to propose new hypotheses about the importance of biobanking promotion which can be tested on larger samples and on the examples of other organizations.

## Conclusion

The present study revealed several important aspects.

First, despite the fairly high level of awareness among the employees of the organizations surveyed about the work of biobanks (almost 90% know what biobanks are), not everyone is aware of the existence of such a structural unit in their organization. The explanation for this fact lies in the organizations’ lack of activity and the insufficient motivation among employees to receive this kind of information on the other.

Secondly, an important factor in evaluating the biobank’s activities, which is the availability of information and samples, is the respondent’s personal experience of cooperation with the biobank. Respondents who have applied to the biobank rate its activities much higher than those who have not applied. They also would like to receive additional information on various aspects of biobank activities.

Third, this study also examined aspects of cooperation with biobanks, such as the personal experience of donation and the willingness (and practice) to involve friends and patients in donation. Respondents who are experienced donors or who plan to become donors in the future are more likely to recommend their patients and acquaintances to donate biospecimens or have already done so. In addition, the experience of donation and patient engagement correlates with the practice of biobanking requests. We concluded that professionals should foster a sense of interest in the development of biobanks, and this can be achieved by creating mechanisms for additional tangible and intangible incentives to attract donors, as well as by encouraging research activities.

Fourth, the majority of respondents are interested in additional information about the activities of biobanks. Lack of awareness among the population was identified as one of the main reasons hindering the development of biobanks. However, it is crucial to comprehend what type of awareness we are discussing. General information is probably no longer sufficient, but more substantial information is needed. Due to the lack of regulation in the industry in Russia, experts show a wide range of opinions regarding the ethical and legal aspects of biobanking. The development of a transparent regulatory framework will facilitate the work of both healthcare professionals and researchers, while also providing additional assurances to potential donors.

Fear of misuse of biospecimens and distrust in scientific research are generally regarded by the public as serious obstacles. Medical professionals who lack sufficient and reliable information have difficulty motivating their patients.

The further development of biobanking therefore requires, on the one hand, additional information for both professionals and the public, and, on the other hand, an increase in the motivation of professionals to cooperate with biobanks in various fields: in scientific research, as donors, and in the involvement of friends and patients in this practice. However, we emphasize that what is needed is not broad general information, but careful work with all interested and involved groups to explain all the nuances of biobanking and to encourage professionals to contact biobanks for samples.

Thus, collaboration among various stakeholders – physicians, researchers, and potential donors is essential. Consolidated efforts from different parties are required, including government support, backing from professional and scientific communities, cooperation with patient organizations and foundations, and the cultivation of a favorable public opinion through media channels and within medical organizations during the trust-based interactions between physicians and patients.

## Data Availability

The raw data supporting the conclusions of this article will be made available by the authors, without undue reservation.

## References

[1] Campbell LD Astrin JJ DeSouza Y Giri J Patel AA Rawley-Payne M. The 2018 revision of the ISBER best practices: summary of changes and the editorial Team’s development process. Biopreserv Biobank. (2018) 16:3–6. doi: 10.1089/bio.2018.0001, PMID: 29393664 PMC5846567

[2] Argudo-Portal V Domènech M. The reconfiguration of biobanks in Europe under the BBMRI-ERIC framework: towards global sharing nodes? Life Sci Soc Policy. (2020) 16:9. doi: 10.1186/s40504-020-00105-3, PMID: 33000342 PMC7528224

[3] De Souza YG Greenspan JS. Biobanking past, present and future: responsibilities and benefits. AIDS. (2013) 27:303–12. doi: 10.1097/QAD.0b013e32835c1244, PMID: 23135167 PMC3894636

[4] Coppola L Cianflone A Grimaldi AM Incoronato M Bevilacqua P Messina F. Biobanking in health care: evolution and future directions. J Transl Med. (2019) 17:172. doi: 10.1186/s12967-019-1922-3, PMID: 31118074 PMC6532145

[5] Zohouri M Ghaderi A. The significance of biobanking in the sustainability of biomedical research: a review. Iran Biomed J. (2020) 24:206–13. doi: 10.29252/ibj.24.4.206, PMID: 32306718 PMC7275812

[6] ElObeid AS AlAbdudlkarim I. The role of biobanks in elucidating prevalent genetic diseases in Saudi Arabia. Drug Discov Ther. (2016) 10:226–33. doi: 10.5582/ddt.2016.01044, PMID: 27594298

[7] Olson JE Bielinski SJ Ryu E Winkler EM Takahashi PY Pathak J. Biobanks and personalized medicine. Clin Genet. (2014) 86:50–5. doi: 10.1111/cge.12370, PMID: 24588254 PMC4898781

[8] Anisimov SV Meshkov AN Glotov AS Borisova AL Balanovsky OP Belyaev VE. National Association of biobanks and biobanking specialists: new Community for Promoting Biobanking Ideas and Projects in Russia. Biopreserv Biobank. (2021) 19:73–82. doi: 10.1089/bio.2020.0049, PMID: 33058731

[9] Litton JE. Launch of an infrastructure for Health Research: BBMRI-ERIC. Biopreserv Biobank. (2018) 16:233–41. doi: 10.1089/bio.2018.0027, PMID: 29781706

[10] Garcia DL Bracci PM Guevarra DM Sieffert N. International Society for Biological and Environmental Repositories (ISBER) tools for the biobanking community. Biopreserv Biobank. (2014) 12:435–6. doi: 10.1089/bio.2014.1264, PMID: 25496158

[11] Decree of the Government of the Russian Federation, (2019) “On approval of the Federal Scientific and technical program for the development of genetic technologies for 2019–2030” (with amendments and additions). Available at: (https://base.garant.ru/72228722/).

[12] Sarmanaev SK Shirokov AY Vasiliev SA Osavelyuk AM Zenin SS Suvorov GN. Proposals for extending the Russian biobanks functions to protect genomic information. Lex Russica. (2019) 6:153–60. doi: 10.17803/1729-5920.2019.151.6.153-160

[13] Domaradzki J Pawlikowski J. Public attitudes toward biobanking of human biological material for research purposes: a literature review. Int J Environ Res Public Health. (2019) 16:2209. doi: 10.3390/ijerph16122209, PMID: 31234457 PMC6617000

[14] Amin L Hashim H Mahadi Z Ismail K. Determinants of the willingness to participate in biobanking among Malaysian stakeholders in the Klang Valley. BMC Med Res Methodol. (2018) 18:163. doi: 10.1186/s12874-018-0619-2, PMID: 30518344 PMC6282379

[15] Domaradzki J Walkowiak D. When biobanks meet religion: association between religiosity and attitudes of polish medical students toward biobanking of human biological material for research purposes. J Relig Health. (2024) 63:1178–213. doi: 10.1007/s10943-023-01932-2, PMID: 37847446 PMC10965646

[16] Heredia NI Krasny S Strong LL Von Hatten L Nguyen L Reininger BM. Community perceptions of biobanking participation: a qualitative study among Mexican-Americans in three Texas cities. Public Health Genomics. (2017) 20:46–57. doi: 10.1159/000452093, PMID: 27926908 PMC5453816

[17] Tsvetkova LA Eritsyan KY Antonova NA. Russian students’ awareness of and attitudes toward donating to biobanks. Psychol Russia. (2016) 9:30–8. doi: 10.11621/pir.2016.0203

[18] Antonova NA Eritsyan KY Tsvetkova LA. Opinion and attitudes of the university community towards biobank donation. Soc Psychol Soc. (2019) 10:169–81. doi: 10.17759/sps.2019100110

[19] Antonova N Eritsyan K. It is not a big deal: a qualitative study of clinical biobank donation experience and motives. BMC Med Ethics. (2022) 23:7. doi: 10.1186/s12910-022-00743-6, PMID: 35090454 PMC8800256

[20] Barnes RO Shea KE Watson PH. The Canadian tissue repository network biobank certification and the College of American Pathologists Biorepository Accreditation Programs: two strategies for knowledge dissemination in biobanking. Biopreserv Biobank. (2017) 15:9–16. doi: 10.1089/bio.2016.0021, PMID: 27740852

[21] Leiman DA Lorenzi NM Wyatt JC Doney AS Rosenbloom ST. US and Scottish health professionals’ attitudes toward DNA biobanking. J Am Med Inform Assoc. (2008) 15:357–62. doi: 10.1197/jamia.M2571, PMID: 18308988 PMC2410009

[22] Alahmad G Al Jumah M Dierickx K. Confidentiality, informed consent, and children’s participation in research involving stored tissue samples: interviews with medical professionals from the Middle East. Narrat Inq Bioeth. (2015) 5:53–66. doi: 10.1353/nib.2015.0013, PMID: 25981282

[23] Caixeiro NJ Byun HL Descallar J Levesque JV de Souza P Soon LC. Health professionals’ opinions on supporting a cancer biobank: identification of barriers to combat biobanking pitfalls. Eur J Hum Genet. (2016) 24:626–32. doi: 10.1038/ejhg.2015.191, PMID: 26328505 PMC4930095

[24] Kamenskikh EM Bakhareva YO Demchenko YD Sokolova TS Kazakov SD Chubakova KA. Awareness of biobanking among patients and doctors: experience of the Tomsk oblast. Cardiovasc Ther Prev. (2023) 22:3678. doi: 10.15829/1728-8800-2023-3678

[25] Lhousni S Boulouiz R Abda N Tajir M Bellaoui M Ouarzane M. Assessment of knowledge, attitudes and support of health professionals towards biobanks in eastern Morocco. Open J Epidemiol. (2019) 9:191–201. doi: 10.4236/ojepi.2019.93016

[26] Kintossou AK N’dri MK Money M Cissé S Doumbia S Soumahoro MK. Study of laboratory staff’ knowledge of biobanking in Côte d’Ivoire. BMC Med Ethics. (2020) 21:88. doi: 10.1186/s12910-020-00533-y, PMID: 32917182 PMC7488401

[27] Gaskell G Gottweis H. Biobanks need publicity. Nature. (2011) 471:159–60. doi: 10.1038/471159a, PMID: 21390108

[28] Buhmeida A Assidi M Alyazidi O Olwi DI Althuwaylimi A Yahya FM. Assessment of biobanking knowledge and attitudes towards biospecimen donation among healthcare providers in Saudi Arabia. Int J Environ Res Public Health. (2022) 19:11872. doi: 10.3390/ijerph191911872, PMID: 36231176 PMC9565163

[29] Abdelhafiz AS Sultan EA Ziady HH Sayed DM Khairy WA. Knowledge, perceptions and attitude of Egyptian physicians towards biobanking issues. PLoS One. (2021) 16:e0248401. doi: 10.1371/journal.pone.0248401, PMID: 33770108 PMC7996976

[30] Fradgley EA Chong SE Cox ME Paul CL Gedye C. Enlisting the willing: a study of healthcare professional-initiated and opt-in biobanking consent reveals improvement opportunities throughout the registration process. Eur J Cancer. (2018) 89:36–41. doi: 10.1016/j.ejca.2017.10.025, PMID: 29223480

[31] Serrano NC Guio-Mahecha E Becerra-Bayona S Luna-González ML Quintero-Lesmes DC. The perception of different social agents in Colombia regarding biobanks for research purposes. Biomedica. (2018) 38:569–76. doi: 10.7705/biomedica.v38i4.4327, PMID: 30653871

[32] Federal Law, (2012) “On blood donation and its components” (with amendments and additions). Available at: (https://base.garant.ru/70204234/).

