# Supplementary material for: Healthcare professionals and scientists’ collaboration with biobanks: a pilot study on the assessment of knowledge and attitudes toward biospecimen donation
Source: Front Med (Lausanne). 2025 Feb 17;12:1497209. doi: 10.3389/fmed.2025.1497209 (PMC11872889; doi:10.3389/fmed.2025.1497209)
Supplement: Supplementary file 1 [file Table_1.DOCX]

Dear colleagues!

Please participate in a survey on biobanking and bioresource collections funded by the grant "Human Reproductive Health." The survey aims to analyze Institute staff's awareness and confidence in biobanking to improve its quality.

The survey takes less than 10 minutes to complete and is anonymous. If desired, you may provide an email address for feedback at the end of the survey.

____________________________________________________________________________

Biobanking Survey

1. Gender

- Man
- Woman

2. Age _______

3. The level of education

- Secondary general education
- Secondary vocational education
- Incomplete higher education
- Bachelor
- Master
- Specialist
- Candidate of Sciences
- Doctor of Sciences

4. Your specialty (according to your diploma) _______

5. Institution

- D.O. Ott Research Institute of Obstetrics, Gynecology and Reproductology, St. Petersburg
- Research Centre for Medical Genetics, Moscow
- Surgut State University, Surgut

6. Job position

- Researcher
- Laboratory assistant
- Physician
- Nursing staff
- Postgraduate, master's student, student
- Medical resident
- Administrative staff
- Other _______

7. Have you ever heard of biobanks?

- Yes
- No
- Don’t know

8. Where have you heard about biobank before?

- At seminar/conference
- Mass media, social networks
- While studying at a university
- Business need
- During informal communication with colleagues
- Don’t know
- Other _______

9. In your opinion, what is a biobank? Please select all that apply.

- Research center
- Collection of biological samples and data
- The organization or department of the organization handling biological samples and data
- Cryostorage
- Museum
- Don’t know

10. What types of research can be conducted using biobank samples?

- Commercial
- Non-commercial
- Don’t know

11. In your opinion, how do samples arrive in the biobank? Please select all that apply.

- The donor specifically donates the material to the biobank
- The biobank receives the material remaining after performing various procedures
- Don’t know

12. Is it possible to do without informed voluntary consent from donors when donating material to a biobank?

- Possible
- Possible in some cases
- Impossible
- Don’t know

13. Have you offered your friends or patients to participate in the donation of biological material to a biobank?

- Yes
- No
- Don’t know

14. Have you ever donated your biological material to the biobank?

- Yes
- No
- Don’t know

15. Are you ready to offer your patients or friends to donate their biological material to a biobank?

- Absolutely ready
- Ready
- Not ready
- Absolutely not ready

16. Are you ready to offer your patients or friends to donate their biological material to a biobank?

- Absolutely ready
- Ready
- Not ready
- Absolutely not ready

17. Do you agree that donors who provide their biological material to a biobank should receive anything?

- Strongly agree
- Agree
- Disagree
- Strongly disagree
- Don’t know

18. If you agree that donors should receive something in return then what? Please select all that apply.

- Monetary reward
- Service (doctor consultation and/or diagnostic test)
- Information about all obtained results of research in which samples from the donor were used
- Don’t know

19. Did you know that there is a biobank in your organization?

- Yes
- No
- Don’t know

20. Have you requested samples or services from your organization's biobank?

- Yes
- No but I plan to
- No and I don't plan to
- Don’t know

21. Are samples from your organization's biobank available to you?

- Fully available
- Partially available
- Completely unavailable
- Don’t know
- Never applied

22. Do you concur that the samples in your institution's biobank hold exceptional scientific merit?

- Strongly agree
- Agree
- Disagree
- Strongly disagree
- Don’t know

23. Rate the availability of information regarding biobanking activities within your organization on a scale.

- 1 - Unavailable
- 2 - Partially available
- 3 - Fully available

24. How much do you trust the biobank and its activities?

- Completely trust
- Rather trust
- Rather distrust
- Completely distrust
- Don’t know

25. What is missing for the development of your organization's biobanks? Please select all that apply.

- Trusting communication between doctor and patient
- Clinicians’ skills to motivate to donate their biological material
- Motivations of medical personnel collecting biological material from donors
- Awareness of biobanking activities among staff of the institution
- Sufficient number of qualified personnel
- Technical capabilities (equipment, etc.)
- Don’t know
- Other _______

26. What, in your opinion, are the obstacles to consent to the donation of biological material from donors/patients? Please select all that apply.

- Health concerns
- Concerns about using biosamples in ways that are not in the patient’s best interest
- Low level of patient awareness of the benefits of biobanking
- No trusting communication between the patient and the doctor
- No offer of remuneration to donor/patient for donation of biomaterial
- Difficulties in understanding voluntary informed consent
- Distrust of scientific research in general
- Don’t know
- Other _______

27. Are you ready to take part in the development of a biobank in your organization?

- Absolutely ready
- Ready
- Not ready
- Absolutely not ready

28. What information would you like to learn more about the biobank in your institution? Please check everything you think is necessary.

- How the biobank works
- What samples are available in the biobank and their number
- How to get samples from the biobank
- How biosamples are stored in the biobank
- How is the biobank equipped
- Don’t know
- Other _______

29. Provide your e-mail address for feedback (optional) _______
